# Human Placental-Specific Epipolymorphism and its Association with Adverse Pregnancy Outcomes

**DOI:** 10.1371/journal.pone.0007389

**Published:** 2009-10-19

**Authors:** Ryan K. C. Yuen, Luana Avila, Maria S. Peñaherrera, Peter von Dadelszen, Louis Lefebvre, Michael S. Kobor, Wendy P. Robinson

**Affiliations:** 1 Department of Medical Genetics, University of British Columbia, Vancouver, British Columbia, Canada; 2 Department of Obstetrics and Gynaecology, University of British Columbia, Vancouver, British Columbia, Canada; University of Georgia, United States of America

## Abstract

Interindividual variation in DNA-methylation level is widespread in the human genome, despite its critical role in regulating gene expression. The nature of this variation, including its tissue-specific nature, and the role it may play in human phenotypic variation and disease is still poorly characterized. The placenta plays a critical role in regulating fetal growth and development in ways that have lifelong effects on health. To identify genes with a high degree of interindividual DNA methylation variation in the human placenta, we surveyed the human genome using the Illumina GoldenGate Methylation Cancer panel targeting 1505 CpG sites of 807 genes. While many sites show a continuous pattern of methylation levels, *WNT2*, *TUSC3* and *EPHB4* were identified to have a polymorphic “on-or-off” pattern of DNA methylation variation at their promoter region which was confirmed by pyrosequencing. Methylation of these genes can be found in 7%–25% of over 100 placentas tested. The methylation state at the promoter of these genes is concordant with mRNA allelic expression. In three informative cases *TUSC3* was observed to be methylated on the maternal allele, and it is thus possible this represents a polymorphically imprinted gene. Furthermore, *TUSC3* promoter methylation showed evidence for association with preeclampsia. A biological significance of these methylation allelic polymorphisms (MAPs) to human placental diversity is further implied by their placental specificity and absence in mouse. An extended study of blood suggests that MAPs may also be found in other tissues, implicating their utility for tissue-specific association with complex disorders. The identification of such “epipolymorphism” in other tissues and their use in association studies, should improve our understanding of interindividual phenotypic variability and complex disease susceptibility.

## Introduction

Gene expression within various human tissues displays interindividual variability that can contribute to phenotypic variation [1]–[3]. Some of this variability is due to DNA sequence differences, such as single nucleotide polymorphism (SNP) and copy number variation (CNV) [4], while environmentally mediated or stochastic effects on epigenetic programming may also affect gene expression [4]. Investigation of monozygotic twins suggests a genetic contribution to gene expression variation [5], [6]; however, non-Mendelian inheritance of allelic variation is also observed [7], [8]. A large-scale analysis of allele-specific gene expression showed that allelic differences in expression level may affect up to 50% of human genes [9]. As only a small fraction of genetic polymorphisms are located in gene regulatory regions, epigenetic variation, that is independent of local sequence changes, may also contribute to a significant portion of variation in gene expression.

DNA methylation is a well-characterized form of epigenetic modification in mammals, and methylation of CpG sites in the promoter regions of genes can critically affect transcriptional regulation [10]. However, evidence for a gene silencing effect of promoter DNA methylation mainly comes from cancer studies, while this relationship in normal tissues has been less clear [11], [12]. Identifying a correlation between gene expression and promoter methylation compared across normal tissues may be confounded by the presence of multiple tissue-specific differentially methylated regions (tDMRs), as well as presence of other tissue-specific regulatory factors that affect the level of expression [4]. Also, some tDMRs exhibit a composite methylation pattern, i.e. a mix of methylated and unmethylated alleles, possibly due to cellular heterogeneity. Even if DNA methylation silences the promoter completely, large changes in gene expression level may not be observed [12]. Thus, identifying distinct DNA methylation differences among individuals within a particular tissue would be useful for demonstrating the regulatory role of DNA methylation on gene expression.

While DNA methylation variation at specific loci, such as imprinted genes, genes on the X-chromosome and transposable elements has been reported [13]–[17], interindividual differences in DNA methylation for other genes in human tissues is less well-studied. A genome-wide study of interindividual DNA methylation variation in the human germline revealed that DNA methylation differences can be established during development [18]. Skewed allelic expression associated with sequence-dependent DNA methylation has also been reported [19]. Further understanding of the extent of tissue-specific methylation variability, its etiology, and its role in affecting gene expression variation is needed.

We hypothesize that sequence-independent effects on DNA methylation set in early development may contribute an additional layer to human phenotypic variation. In order to identify distinct DNA methylation differences between individuals and assess the regulatory role of DNA methylation on gene expression and phenotypic variation, we surveyed the human genome using the Illumina GoldenGate Methylation Cancer Panel I. We chose to study placenta as it plays a vital role in human health due to its essential role in regulating fetal growth and development and the long term consequences of *in utero* development on disease in adulthood [20]. In addition, placenta has been reported to have high variability in overall DNA methylation compared to other tissues as investigated by the same Illumina methylation array [21], and increased epigenetic variability in the placenta may have evolved in response to its role in mediating the conflicting demands of mother and fetus [22]. Although the Illumina GoldenGate Methylation Cancer panel I targets mainly cancer-related genes, the pseudomalignant nature of the placenta makes it suitable for this study [23], [24].

## Results

### Identifying genes with “on-or-off” polymorphic DNA methylation

**Figure 1:**
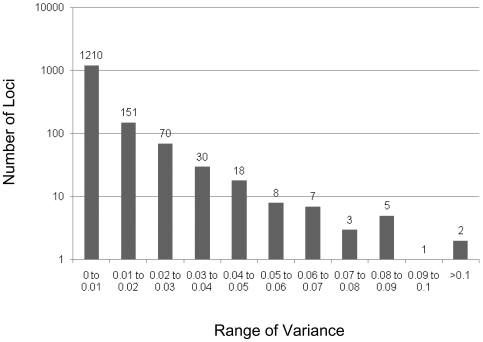
Frequency distribution of DNA methylation variances for 1505 CpG sites in 13 normal placental samples. The average variance is 0.007. The value for 1.5 SD above the mean variance is 0.025. There are 106 CpG sites with variance greater than 1.5 SD.

**Figure 2:**
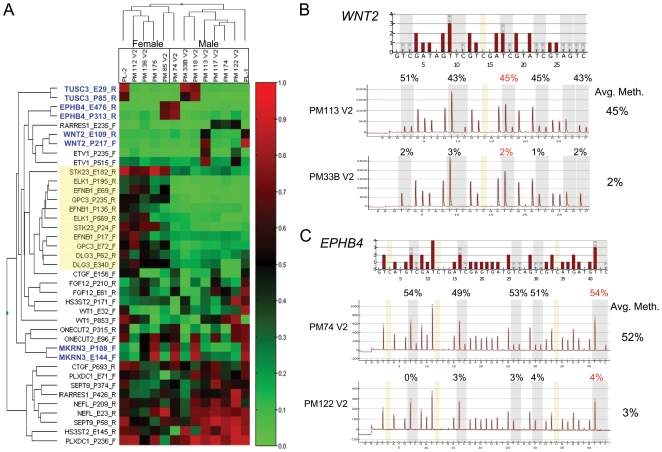
Genes exhibiting high interindividual variance in methylation values in the human placentas. (A) Heat-map of 19 genes with at least 2 probes having methylation variance greater than 1.5 SD from the mean. Probes and sample names are shown and with hierarchical clustering of beta values based on 1-r (Illumina Beadarray software). A beta value of zero (indicated in bright green) represents an unmethylated locus and one (indicated in bright red) represents a methylated locus. Probes for genes on the X chromosome are highlighted by a yellow box and the probes being further investigated here are bolded in blue. (B and C) Validation of variable methylation by bisulfite pyrosequencing for (B) *WNT2* and (C) *EPHB4*. CpG sites that are targeted by the Illumina probes are highlighted in red. One methylated sample and one unmethylated sample are shown for each gene. Reference pyrograms are shown on top.

Using the Illumina GoldenGate methylation Beadarray, we initially investigated DNA samples from whole villi (fetus side) of 13 normal placentas (5 female and 8 male) without pregnancy complication. To identify probes (CpG sites) that have distinct classes of DNA methylation levels among placentas, we first calculated the variance of the β-value (proportional to level of DNA methylation) for each probe. The majority of sites 1210 of 1505, showed very little variability (variance<0.01) (Figure 1) and these were generally either always methylated or always unmethylated. However, the distribution of variances has a broad tail and many sites showed extremely variable methylation patterns. While not all CpG sites associated with a single gene necessarily are expected to be methylated similarly, to reduce the probability of variability due to technical artefact or to SNPs in the associated primer sequences, we identified genes for which at least 2 associated CpG sites demonstrated a β-value variance greater than 1.5 SD from the mean variance for all samples. Using this criterion, 19 out of 576 genes that had probes targeting two or more CpG sites were identified as having highly variable DNA methylation among individual placentas (Figure 2A). Among these 19 genes, 14 genes are located on the autosome while 5 are on the X chromosome. As expected, methylation at these X-linked sites (all in gene promoter regions) correlates with sex of the placental sample (i.e. higher methylation in female than in male) given that promoter DNA methylation is enriched on the inactive X chromosome of females [25]. Detection of additional X-linked genes was limited by our strict criteria for this screen (i.e. two sites, both >1.5 SD above the mean). *WT1*, an imprinted gene with polymorphic imprinting in placenta [26], was detected, which further validates this approach. Variable DNA methylation identified at another imprinted gene, *MKRN3* (Figure S1A), suggests it may also be polymorphically imprinted in placenta.

We chose three autosomal genes which had the most concordant methylation patterns between the two associated CpG sites assayed for further follow-up: *WNT2*, *EPHB4*, and *TUSC3* (Figure 2A). These genes also appeared to have a bimodal distribution of methylation suggestive of an on/off switch. The methylation pattern for these genes was confirmed and quantified more accurately by gene-specific bisulfite pyrosequencing using primers without any known SNP or CpG site bias (Figure 2B, Figure S1B). A similar methylation level was found for every CpG investigated within the sample group with around 50% methylation in “methylated” cases and almost no methylation in “unmethylated” cases (Figure 2B, Figure S1B). No within-placenta variability was observed as different sites sampled from the same placenta always displayed concordant methylation levels (Figure S2). We further investigated samples from more than 100 placentas by the Illumina array (49 placentas run on separate Beadarrays than the original set) and bisulfite pyrosequencing (all placentas). Using the same threshold to search for distinct methylation polymorphism, 12 genes met the criteria in the Illumina methylation analysis of the additional 49 placental samples (Figure S3A). Nine out of the 12 genes, including *TUCS3* and *WNT2*, were in common with those found in the initial analysis of 13 placental samples. Although *EPHB4* did not meet the variance cut-off observed in the initial set, distinct polymorphic methylation was observed with 3 of the 49 samples exhibiting a “methylated” state (Figure S3B). The lower variance was thus a consequence of the lower frequency of “methylated” alleles in the larger sample set. All samples for the initially identified three CpGs could be classified as “methylated” or “unmethylated” (i.e. the distribution of values was again distinctly bimodal) and the methylation frequency (ratio of number methylated cases to total number cases) for these genes ranged from 0.07 to 0.25 (Table 1).

**Table 1:** Correlation between MAP and clinical status.

Gene Name	M/U	Total	MF	MA (year)	GA (week)	BW (g)	Gender (M∶F)	IUGR	No IUGR	*p*-value	EOPET	LOPET	No PET	*p*-value
***EPHB4***	M	9	0.07	33.0	36.9	2878.3	4∶5	2	7	1.00	1	1	7	1.00
	U	115		34.6	36.9	2802.0	56∶59	30	85		15	17	83	
***TUSC3***	M	31	0.25	34.5	36.2	2776.7	15∶16	10	21	0.48	3	9	19	<0.05
	U	91		34.3	37.1	2836.3	48∶43	22	69		13	9	69	
***WNT2***	M	25	0.20	33.6	37.7	3100.7	12∶13	3	22	0.08	1	4	20	0.37
	U	97		34.7	36.6	2715.5	46∶51	29	68		15	14	68	

M = Methylated; U = Unmethylated; MF = Methylation frequency (Number of methylated cases/Total number of cases); MA = Maternal age; GA = gestational age; BW = birth weight; EOPET = early onset preeclampsia (<34 weeks' gestation); LOPET = late onset preeclampsia (≥34 weeks' gestation).

### Correlation of DNA methylation and gene expression

**Figure 3:**
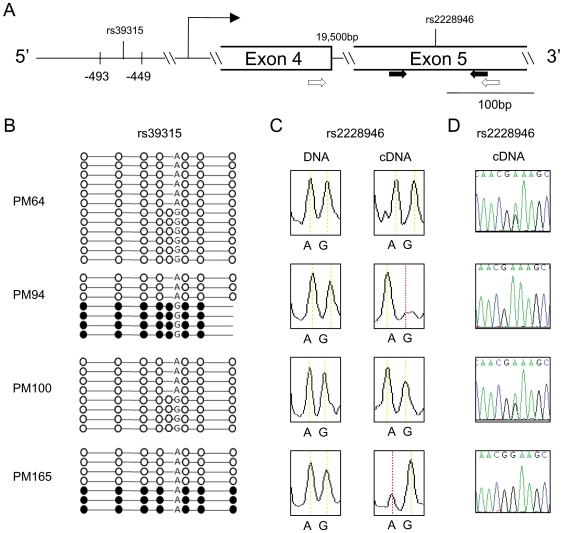
Allele-specific DNA methylation and mRNA expression of *WNT2*. (A) Schematic of the *WNT2* locus showing the regions investigated by clonal bisulfite pyrosequencing of the promoter (−493 to −449 relative to the transcriptional start site according to NM_003391) and genotyping assays within exon 5. PCR primers for DNA and cDNA genotyping by iPlex are indicated by black arrows while RT-PCR primers for mRNA genotyping are indicated by arrows highlighted in white. (B) Bisulfite pyrosequencing of single clones from four placental samples. The A/G polymorphism of SNP rs39315 is indicated. Each row represents one clone and each circle represents one CpG. Methylated CpGs are shown in black while unmethylated CpGs are shown in white. The presence of a cytosine proximal to this A/G SNP site creates a polymorphic CpG site. (C) Allele-specific expression of *WNT2* based on the analysis of the A/G allele of rs2228946 in DNA and cDNA by iPlex. Peak height of the alleles corresponds to the relative amount of alleles present in the sample. (D) Validation of allele-specific expression of *WNT2* by cDNA-specific primers. Double peaks are observed in unmethylated samples while single peaks are found in methylated samples.

**Figure 4:**
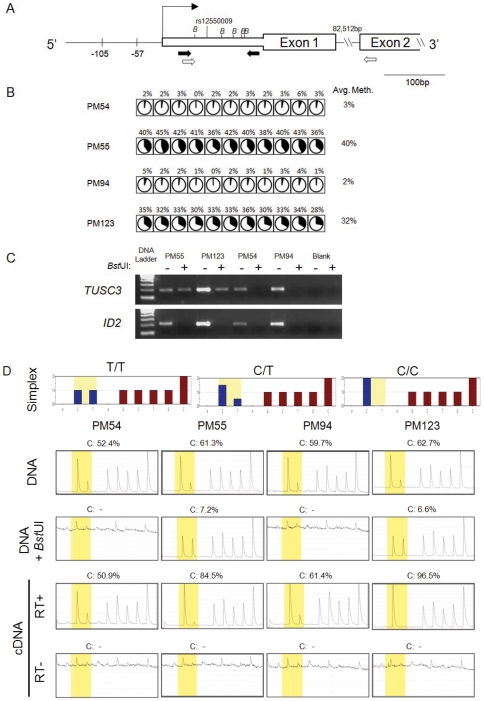
Promoter CpG methylation correlates with lack of *TUSC3* gene expression. (A) Schematic of *TUSC3* locus showing the regions investigated by bisulfite pyrosequencing on the promoter (−105 to −57 relative to the transcriptional start site according to NM_006765) and genotyping assays of the 5′ untranslated region. PCR primers for DNA genotyping are indicated by black arrows while RT-PCR primers for mRNA genotyping are indicated by arrows highlighted in white. Enzyme recognition sites for *Bst*UI are indicated by “*B*”. (B) Methylation status of *TUSC3* promoter region studied by bisulfite pyrosequencing. A similar methylation level of every CpG within each sample is observed and the gene follows “on-or-off” methylation pattern. Each circle represents a CpG site in a sample. Area shaded in black is proportional to the methylation level of the CpG site indicated by pyrosequencing. (C) Validation of complete methylation-sensitive restriction enzyme digestion on unmethylated molecules. Genomic DNA was predigested with *Bst*UI followed by PCR amplification with *TUSC3* and *ID2* specific primers (Table S2). *Bst*UI digestion sites within the *ID2* region were unmethylated (Figure S6) and, therefore, no PCR product was generated after enzyme digestion. (D) Allele-specific methylation of *TUSC3* on the fragment containing SNP rs12550009 demonstrated by enzyme digestion pyrosequencing. The “Simplex” diagrams (top) show the reference pyrograms by genotype. A heterozygous CT in the methylated samples (PM55 and PM123) displays a homozygous T pattern after *Bst*UI digestion indicating predominant methylation of the T allele. Allele-specific mRNA expression is concordant with allele-specific methylation on the same SNP rs1250009. Predominant expression of C alleles was observed in the cDNAs generated by RNA specific primers (bottom). RT+ and RT− represent assays with Reverse Transcriptase and without Reverse Transcriptase, respectively.

Since the average methylation level of the CpGs in those cases classified as “methylated” was close to 50% based on bisulfite pyrosequencing, we speculated that the DNA methylation may cause allele-specific variation in gene expression. Therefore, heterozygous SNPs in the coding regions of these genes were identified and the genotype of DNA and cDNA extracted from the same placenta were compared by primer extension assay (Figure 3, Figure S4). Clonal bisulfite pyrosequencing of the *WNT2* promoter demonstrated monoallelic DNA methylation in the methylated cases (Figure 3B). Furthermore, biallelic gene expression was observed in the unmethylated cases, while monoallelic expression was found in the methylated cases (Figure 3C, Figure S4). A similar observation was found for *EPHB4* (Figure S5). To determine the relationship between promoter DNA methylation and gene expression, we identified four cases heterozygous for SNP rs12550009 located within the 5′ UTR of *TUSC3* (Figure 4A). Methylation-sensitive enzyme digestion followed by pyrosequencing genotyping revealed allele-specific methylation of the “T” alleles in the two of these cases which were methylated at this gene (Figure 4D). Genotyping cDNA with pyrosequencing using RNA-specific RT-PCR primers demonstrated biallelic expression in the unmethylated cases while the “C” alleles were predominantly expressed in the methylated cases. Thus, lack of DNA methylation of the gene promoter is correlated with gene expression. Unlike the sequence-dependent allele-specific DNA methylation described in another study [19], the present polymorphic DNA methylation had no correlation with the genotypes of the SNPs (Table S1). To distinguish this on-off type of epigenetic polymorphism, we suggest a term called Methylation Allelic Polymorphism (MAP). This term can generally be used to apply to any polymorphic methylation, including that attributable to imprinting or local sequence effects, as well as that due to other causes (stochastic, environment etc).

### Correlation between MAP and pregnancy complication

Intriguingly, the genes exhibiting MAP identified here are highly expressed in the placenta [27]. Furthermore, *WNT2* and *EPHB4*, are crucial for placenta development [28]–[30]. The variable allelic gene expression caused by MAP may have functional consequences to placental physiology. In particular, the expression of *TUSC3* was downregulated in trophoblast upon hypoxic (a characteristic feature in preeclampsia) *in vitro* culturing [31]. To determine whether there is a correlation between MAP and pregnancy disorders, the studied samples were categorized according to the presence or absence of intrauterine growth restriction and/or preeclampsia (Table 1). We found a significant difference in DNA methylation frequency of *TUSC3* between normal and preeclamptic pregnancies (Table 1). Specifically, *TUSC3* promoter methylation was found more frequently in the late-onset preeclampsia than normal placentas (*P* = 0.02; Fisher's test). There was no significant correlation of MAP with maternal age, gestational age, and fetus gender or birth weight (Table 1).

### No conservation of MAP in *Ephb4*, *Tusc3* and *Wnt2* of mice

**Figure 5:**
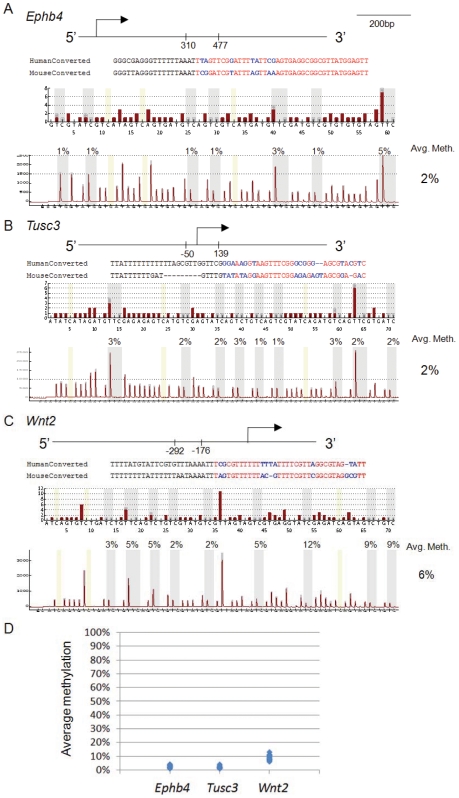
DNA methylation status of MAP conserved regions in mouse. Schematic of (A) *Ephb4* locus (310 to 477 relative to the transcriptional start site according to NM_010144), (B) *Tusc3* locus (−50 to 139 relative to the transcriptional start site according to NM_030254) and (C) *Wnt2* locus (−292 to −176 relative to the transcriptional start site according to NM_003391), showing the regions investigated by bisulfite pyrosequencing. Sequence alignments on bisulfite converted DNA between human and mouse at the first 60 nucleotides including the sequencing primers are shown. Sequences highlighted in red are the nucleotides being investigated while the nucleotides highlighted in blue are the differences between them. Reference pyrograms are provided and one representative sample for each locus is shown. (D) Summary of methylation level at *Ephb4*, *Tusc3* and *Wnt2* in 21 outbred mice. No “on-or-off” methylation variation is found in the mouse conserved regions.

As we observed no cases exhibiting 100% methylation for any of these analyzed sites, the MAP is likely regulated in a specific manner. In order to better understand the regulatory mechanism as well as the functional effect of MAP, we investigated the methylation status of these genes in mice, for which embryonic lethality has been reported in *Wnt2* and *Ephb4* knock-outs [28], [29]. However, the conserved regions of the three genes were unmethylated in the placentas of 21 outbred mice (Figure 5), suggesting MAP in these genes may not be conserved in rodent placenta and implicating a discrepancy of interindividual variation of these genes between human and mouse placentas. Further analysis of MAP in other placental mammals would be interesting to find out if MAP is unique to human placentas.

### Tissue-specificity of MAP

**Figure 6:**
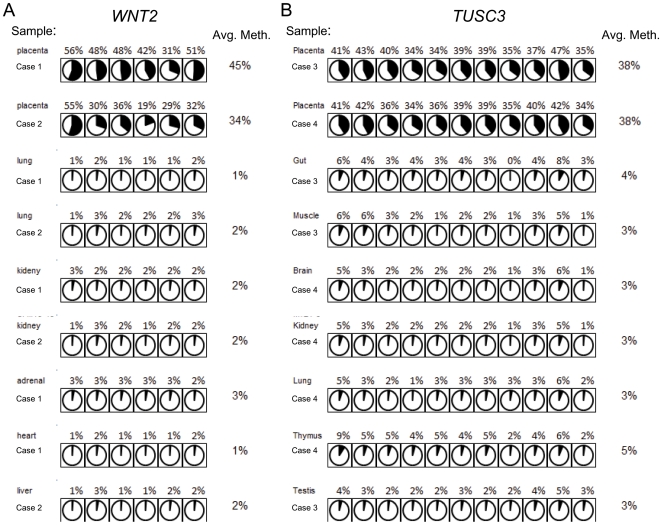
Tissue-specific DNA methylation of *WNT2* and *TUSC3*. (A) DNA samples from two independent fetuses associated with placental methylation at the *WNT2* promoter were investigated by bisulfite pyrosequencing. None of the tissues (lung, kidney, adrenal, heart and liver) other than placenta was methylated. (B) DNA samples from two independent fetuses with placental methylation at the *TUSC3* promoter were investigated by bisulfite pyrosequencing. None of the tissues (lung, kidney, gut, muscle, brain, thymus and testis) other than placenta was methylated. Each circle represents a CpG site in a sample. Area shaded in black is proportional to the methylation level of the CpG site indicated by pyrosequencing.

To determine the tissue specificity of MAP in human, the fetal tissues of abortuses with DNA methylation of *TUSC3* and *WNT2* in the associated placentas were studied. DNA methylation in the promoter of *TUSC3* and *WNT2* was not observed in any of 10 fetal tissues other than placenta (Figure 6). Also, there was no methylation in the maternal blood cells from women carrying placentas with DNA methylation of the *TUSC3* gene (Figure S7A). Even within the methylated placenta, trophoblastic chorionic villi was the only tissue methylated (Figure S7B).

We further tested the genome-wide DNA methylation patterns in blood cells of 18 normal individuals by the Illumina methylation array. Using the same criteria as we analyzed in placentas, 15 genes have highly variable methylation in two associated CpGs (defined as >1.5 SD above the mean) (Figure S8A). 14 of the identified genes were located on the X chromosome, indicating that blood cells are less variably methylated than placenta, which is consistent with a previous study [21]. As expected, we found no MAP in *EPHB4*, *TUSC3* and *WNT2* (Figure S8B). Two distinct CpGs associated with *TRIP6* genes were identified with highly variable methylation, while CpGs associated with two other genes, *NOD2* and *ALOX12*, nearly met these criteria. While the variation was not distributed in a clearly bimodal fashion, the levels of methylation for each pair of CpGs showed a very high degree of correlation, suggesting this is not methodological (measurement error or sequence variants directly affecting probe binding). The variable methylation at *NOD2* was further confirmed by pyrosequencing (Figure S8C). A high degree of allelic variation of *NOD2* expression has been reported elsewhere [32], suggesting this variable methylation reflects this variable expression. As whole blood consists of a mixture of various types of cells, distinct on/off methylation patterns confined to a specific cell type may appear to be continuously distributed due to confounding by the varying proportions of cells among individuals. Analysis of individual blood cell populations would be necessary to determine if this is the case for these genes.

## Discussion

Understanding the source of phenotypic variation among individuals is a fundamental aspect of human biology. Current studies mainly focus on searching for genetic sequence variation which might miss the important phenotypic effects exerted by epigenetic polymorphisms. A study of the *MHC* locus on chromosome 6 in 7 human tissues across 32 individuals showed that around half of the studied loci had some interindividual variability for DNA methylation in at least one tissue [33]. However, this was not extensively quantified and its effect on gene expression was not investigated. Other loci with variable DNA methylation have also been found recently [18], [19], but most, if not all, are dependent on DNA sequence variation within the differentially methylated region. In this report, we identify tissue-specific DNA methylation polymorphisms that can be found in as many as 25% of individuals and cannot simply be explained by the DNA sequence differences generated by common flanking SNPs. They are sequence-independent epigenetic polymorphisms that can act as a *cis*-acting regulator of gene expression.

The silencing effect of DNA methylation on single allele in *EPHB4*, *TUSC3* and *WNT2* resembles the characteristic of imprinted genes. With limited parental DNA and RNA samples from the “methylated” cases, we were unable to rule out the possibility that the genes with MAP are novel polymorphic imprinted genes. Polymorphic imprinting has been reported in humans for *IGF2R* (paternal or biallelic expression) [34] and *WT1* (maternal or biallelic expression) [26]. By screening 70 maternal-fetal pairs for rs1250009 in *TUSC3*, we identified three cases informative for parental origin of the methylation and all three were methylated on the paternal allele. Similarly we identified one “methylated” case of *EPHB4* with paternal expression suggesting the maternal allele was methylated in this case. While the MAPs were maternally methylated in all informative cases we identified, we cannot rule out the possibility that this happened only by chance due to the small sample size. Of the roughly 80 imprinted loci identified to date, few are imprinted in human but not in mouse [35].Thus these results could be consistent with an abnormal or stochastic failure of erasure of this “imprint” in the trophoblastic villi or specifically to a failure to erase a maternal methylation mark.

Alternatively, there may be a lineage-specific acquisition of DNA methylation by a *de novo* mechanism early in development. In this case, either allele could be methylated, or there may be a preference for acquiring methylation on one parental allele due to other epigenetic marks differentiating the two parental chromosomes. It is possible that a random acquisition of DNA methylation on single allele of these genes reflects a selection for reduced expression of these genes which may be relevant to the generation of imprinted genes during evolution [36]. Further investigation of parental origins of the allelic methylation is needed to test this hypothesis.

The fact that none of the cases in this study has complete methylation on both alleles suggested that the regulation of developmental important genes by MAP in placenta is functionally significant. The correlation of *TUSC3* promoter methylation with preeclampsia, a pregnancy disorder that is complicated by placental hypoxia implies a biological relevance to MAP. *TUSC3* is an ortholog of the yeast *Ost3* protein which catalyzes the transfer of an oligosaccharide chain on nascent proteins in the process of N-glycosylation [37]. While the function of *TUSC3* in placenta is unknown, its paralog, *MAGT1*, is believed to be associated with embryonic implantation and hypertension [38]. In addition, *TUSC3* is highly expressed in the human placenta, but expression was reduced after *in vitro* hypoxic culturing of trophoblast [31]. These observations suggest that *TUSC3* may be important in the development of preeclampsia. Further studies are necessary to confirm this association and to identify the intrinsic function of *TUSC3* in the human placenta and its relation to preeclampsia development. Although the clinical status of the placentas did not appear to be related to the methylation pattern of *EPHB4* and *WNT2* in human, a phenotypic effect of these MAP genes on the human placenta cannot be excluded as only two clinical features, IUGR and preeclampsia were evaluated.

The discrepancy of DNA methylation profile between human and mouse might also suggest an evolutionary role. Several DNA methylation studies of placenta revealed a number of tumor-related genes specifically methylated in the human placenta [23], [24]. It is believed that the difference in DNA methylation profile between rodents and primates may account for the disparity of placentation, such as different degree of trophoblast invasiveness, between species. Intriguingly, *EPHB4* and *WNT2* were found to be responsible for vascularisation of placenta which associated with the invasion of spiral arteries [28], [30]. It is possible that the DNA methylation polymorphism in these genes causes a subtle difference in the degree of trophoblast invasiveness among individual human placentas. Many of the other genes detected in our initial screen likewise may play an important role in placentation (Figure 2). For example *CTGF* is an important regulator of VEGF, a factor critical in vascularisation of the placenta and decidua [39]. However, biological effects may be difficult to discern when considering only the methylation status of individual genes as it may be the combined effects of multiple genes that is critical in development of traits, which may explain the marginal significance of disease association for *TUSC3*. The identification of MAP in other genes could be tested for association with complex traits by whole-epigenome association studies [40], [41].

Recently, “epimutation” has been found for *MLH1* and *MSH2* in cancer patients [42], [43]. Similar to the MAP identified here, epimutation can silence the genes in an allelic-specific manner. The distinction is that MAP is more frequent and appears to be set early in development, as we observed no within-placenta heterogeneity and found MAP even in first-trimester placentas (Data not shown). Although additional biological effects of such “epipolymorphism” in human remains to be determined, the functional consequence of imbalanced allelic gene expression is substantial [44], [45]. A genome-wide study of gene expression found that the variation of gene expression between alleles is common in human and it is believed to be the basis for variation in the transmission of some diseases [9], [32]. Thus the study of MAP as a method of identifying allelic expression differences, through measures at the DNA level, should open up a new dimension for future disease association studies. The Illumina methylation array used in this study only targets 807 genes, of which we only considered the limited set of those with multiple CpGs exhibiting correlated methylation patterns. Looking at these same genes more exhaustively, and considering the more than 20,000 genes in the human genome, there should be many more genes identified with MAP which might contribute to the disease susceptibility in a multifactorial and tissue-specific way. The future study of MAP is important for our understanding of interindividual phenotypic variability, as well as complex disease susceptibility.

## Materials and Methods

### Sample collection

This study was approved by the ethics committees of the University of British Columbia and the Children's & Women's Health Centre of British Columbia. Samples from 128 placenta were collected from Vancouver BC Children's & Women's Hospital with informed consent from individuals. Clinical information was collected on prenatal findings, pregnancy complications and birth parameters (gestational age, sex, birth weight etc). Preeclampsia was defined as at least two of the following: (1) hypertension (systolic blood pressure ≥140 mmHg and/or diastolic blood pressure ≥90 mmHg, twice, >4 h apart) after 20 weeks, and proteinuria defined as ≥0.3 g/d or ≥2+ dipstick proteinuria after 20 weeks, (2) non-hypertensive and non-proteinuric HELLP syndrome or (3) an isolated eclamptic seizure without preceding hypertension or proteinuria. Intrauterine growth retardation (IUGR) was defined as either (1) birth weight <3rd percentile for gender and gestational age using Canadian charts, or (2) birth weight <10th percentile with either: (a) persistent uterine artery notching at 22+0 to 24+6 weeks gestation, (b) absent or reversed end diastolic velocity on umbilical artery Doppler, and/or (c) oligohydramnios (amniotic fluid index <50 mm). At least two sites were sampled from each placenta. DNA was extracted and RNAlater (Qiagen) was added for follow up RNA extraction. First-trimester normal placental tissues, peripheral blood samples from normal individuals and fetal tissue biopsies from abortuses were obtained with review board approval and were anonymous to individual identifiers. Outbred mouse placental tissues were obtained from pregnant mice with institutional animal ethics approval.

### DNA methylation analysis

Bisulfite modification of 500 ng of genomic DNA was performed using the EZ DNA Methylation Kit (Zymo Research) according to the manufacturer instructions. After bisulfite treatment, DNA samples were subjected to the Illumina GoldenGate methylation Cancer Panel I array-based assay, using Illumina-supplied reagents and conditions. A β-value of 0 to 1 was reported for each CpG site, signifying the percentage of methylation, from 0% to 100%. β-values were calculated by subtracting background with use of negative controls on the array and taking the ratio of the methylated signal intensity to the sum of both methylated and unmethylated signals.

To identify genes with the most highly variable distribution of methylation values, the variance of β-values among placentas was calculated for each CpG site, as well as the standard deviation of this value relative to the mean variance observed for all CpGs. Those sites with variance values >1.5 standard deviation from the mean were considered to be “highly variable”. To then select for findings not likely to be due to artefact (such as variable hybridization or local sequence variants), only genes with at least two associated highly variable CpGs were considered. The identified pairs of highly variable CpGs associated with the same gene tended to show a good degree of correlation of methylation values and several appeared to have a bimodal distribution in methylation values suggestive of on/off methylation. Four autosomal genes which had the highest correlation in methylation values between the two associated CpGs were selected for follow-up confirmation. Methylation-unbiased PCR and sequencing primers were designed based on the sequences from Illumina probes on the CpG site (Table S2). Pyrosequencing was performed on a Biotage PSQ HS96 Pyrosequencer and the quantitative levels of methylation for each CpG dinucleotide were evaluated with Pyro Q-CpG software (Biotage). A test run for each assay was performed in triplicate to confirm reproducibility. For clonal bisulfite pyrosequencing, PCR product from individual samples was generated by non-biotinated primers (Table S2) and subsequently TA-cloned into pGEM-Teasy vector (Promega). Individual clones were picked and analyzed by pyrosequencing as described.

### SNP genotyping

Multiplex genotyping on genomic DNA was performed by iPlex (Sequenom) in Quebec Genome Centre. Primer sequences for individual SNP genotyping are available upon request. The primer extended products were analyzed and the genotypes determined by mass spectrometric detection using the MassARRAY Compact system (Sequenom). For *Bst*UI predigestion assay followed by pyrosequencing on *TUSC3*, 200 ng of genomic DNA was digested with 100 units of *Bst*UI (New England Biolabs) for 18 hours. 20 ng was used for PCR and *ID2* was used as internal control for validation of complete enzyme digestion in each sample. Pyrosequencing was performed on a Biotage PSQ HS96 Pyrosequencer and the relative levels of allele for the SNP were evaluated with PSQ96MA SNP analysis software (Biotage). Genotyping on mRNA was carried out either with cDNA prepared using Omniscript Reverse Transcriptase Kit (Qiagen) followed by iPlex (Sequenom) or one step RT-PCR (Qiagen) followed by sequencing or pyrosequencing. Primers for the one step RT-PCR assays were designed to span at least one intron (Table S2). PCR without reverse transcriptase was performed on each sample to confirm no genomic DNA contamination.

### Statistical Analysis

All the statistical analysis in this study was performed using VassarStats (http://faculty.vassar.edu/lowry/VassarStats.html).

## Supporting Information

Figure S1 Validation of variable methylation at (A) MKRN3 and (B) TUSC3 by pyrosequencing. Pyrograms from one methylated sample and one unmethylated sample are shown. Reference pyrograms are shown on top. Methylation level of CpG sites that are targeted by the Illumina probes are highlighted in red.
(0.03 MB GIF)

Figure S2 Intraindividual variability of DNA methylation on MAP. DNA methylation level of (A) TUSC3, (B) EPHB4 and (C) WNT2 is given for multiple whole villous samples taken from the same placenta (four sites for PM55, two sites for PM94). On-or-off DNA methylation pattern was consistent from different samples within the same placenta. Each circle represents a CpG site within a sample. The area shaded in black is proportional to the methylation level of the CpG site indicated by pyrosequencing.
(0.08 MB GIF)

Figure S3 Genes exhibiting high interindividual variance in methylation values in a large population of human placentas. (A) Heat-map of 12 genes with at least 2 probes having methylation variance greater than 1.5 SD from the mean. Probes and sample names are shown and with hierarchical clustering of beta values based on 1-r (Illumina Beadarray software). A beta value of zero (indicated in bright green) represents an unmethylated locus and one (indicated in bright red) represents a methylated locus. Probes for genes on the X chromosome are highlighted by a yellow box. (B) Heat-map of EPHB4 in 49 human placentas.
(0.14 MB GIF)

Figure S4 Allele-specific mRNA expression in WNT2. (A) Schematic of WNT2 locus showing the regions investigated by genotyping assays within exon 5 of 3 methylated samples and 3 unmethylated samples. PCR primers for DNA and cDNA genotyping by Sequenom are indicated by black arrows. (B) Allele-specific expression of WNT2 is observed based on the A/G allele of rs2228946 in DNA and cDNA by iPlex. Peak height of the alleles corresponds to the relative amount of alleles present in the sample.
(0.04 MB GIF)

Figure S5 Allele-specific methylation and mRNA expression in EPHB4. (A) Schematic of the EPHB4 locus showing the regions investigated by clonal bisulfite pyrosequencing on the exon 1 (58 to 153 relative to the transcriptional start site according to NM_004444) and genotyping assays for exon 10. PCR primers for DNA by sequencing are indicated by black arrows while RT-PCR primers for mRNA genotyping are indicated by arrows highlighted in white. (B) Bisulfite pyrosequencing of single clones from four placenta samples. Each row represents one clone and each circle represents one CpG. Methylated CpGs are shown in black while unmethylated CpGs are shown in white. (C) Allele-specific expression of EPHB4 at A/G allele of rs314359 in DNA and cDNA by sequencing. Double peaks are shown in unmethylated samples. However, peaks on the SNP were skewed only in cDNA from methylated samples.
(0.09 MB GIF)

Figure S6 CpG methylation status of ID2 on BstUI digestion sites. Schematic of ID2 locus showing the regions investigated by bisulfite pyrosequencing is shown on top (8 to 186 relative to the transcriptional start site according to NM_002166). PCR primers for bisulfite pyrosequencing are indicated by black arrows. Enzyme digestion sites of BstUI are indicated by “B”. Reference pyrogram is provided. CpG sites were unmethylated for this region of ID2 in the 4 samples investigated.
(0.03 MB GIF)

Figure S7 Tissue-specific DNA methylation of TUSC3. (A) DNA samples from two independent fetuses with placentas unmethylated in TUSC3 promoter were investigated by bisulfite pyrosequencing. None of the cases were methylated in other tissues. (B) DNA samples from 3 independent placentas with trophoblastic villi methylated in TUSC3 promoter were investigated by bisulfite pyrosequencing. None of the tissues (Aminon, chorion, cord, decidua and maternal blood) other than whole villi was highly methylated. Each circle represents a CpG site in a sample. Area shaded in black is proportional to the methylation level of the CpG site indicated by pyrosequencing.
(0.12 MB GIF)

Figure S8 Interindividual variance of methylation values in the human blood cells. (A) Heat-map of 14 genes with at least 2 probes having methylation variance greater than 1.5 SD from the mean. Probes and sample names are shown and with hierarchical clustering of beta values based on 1-r (Illumina Beadarray software). A beta value of zero (indicated in bright green) represents an unmethylated locus and one (indicated in bright red) represents a methylated locus. Probes for genes on the X chromosome are highlighted by a yellow box. (B) Heat-map of EPHB4, TUSC3, WNT2 and NOD2 (CARD15) in human blood generated by Illumina GoldenGate Methylation Array. (C) Validation of variable methylation of NOD2 by bisulfite pyrosequencing. Schematic of NOD2 locus showing the regions investigated by bisulfite pyrosequencing (−323 to −266 relative to the transcriptional start site according to NM_022162). One methylated sample and one unmethylated sample are shown. Reference pyrograms are shown on top. Methylation level on CpG sites that targeted by the Illumina probe is highlighted in red.
(0.10 MB GIF)

Table S1 Genotypes at single nucleotide polymorphisms associated with EPHB4,TUSC3, and WNT2 show no association with methylation status of the identified MAP for the same gene.
(0.03 MB XLS)

Table S2 PCR primers and conditions for all assays.
(0.03 MB XLS)
