# Supplementary figures and images for: Human Placental-Specific Epipolymorphism and its Association with Adverse Pregnancy Outcomes
Source: PLoS One. 2009 Oct 19;4(10):e7389. doi: 10.1371/journal.pone.0007389 (PMC2760756; doi:10.1371/journal.pone.0007389)

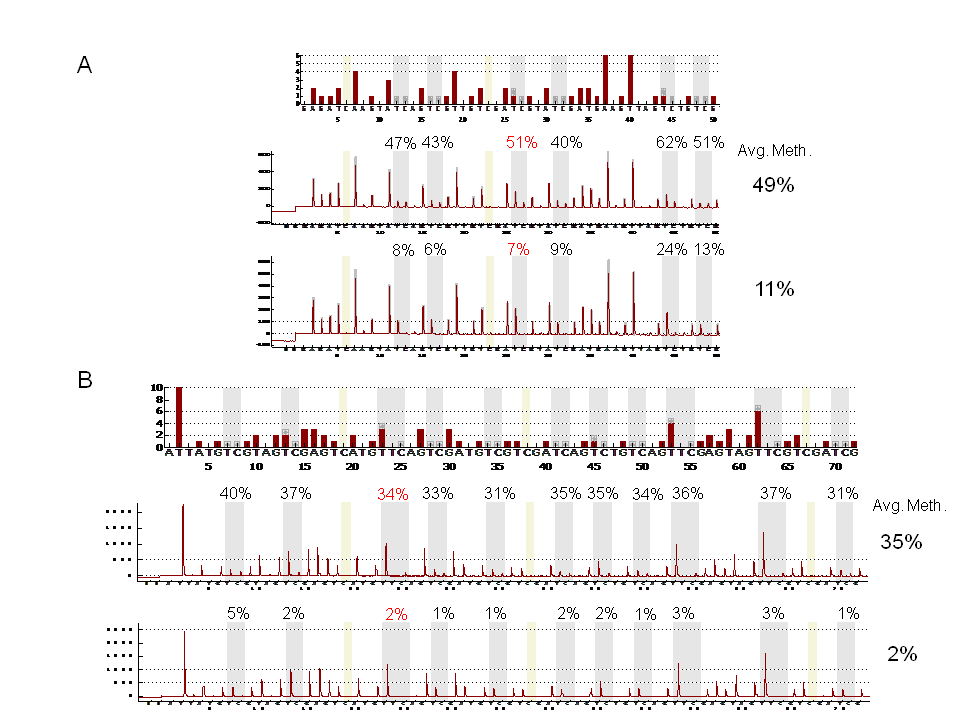

Supplement: Figure S1 — Validation of variable methylation at (A) MKRN3 and (B) TUSC3 by pyrosequencing. Pyrograms from one methylated sample and one unmethylated sample are shown. Reference pyrograms are shown on top. Methylation level of CpG sites that are targeted by the Illumina probes are highlighted in red. (0.03 MB GIF) [file pone.0007389.s001.gif]

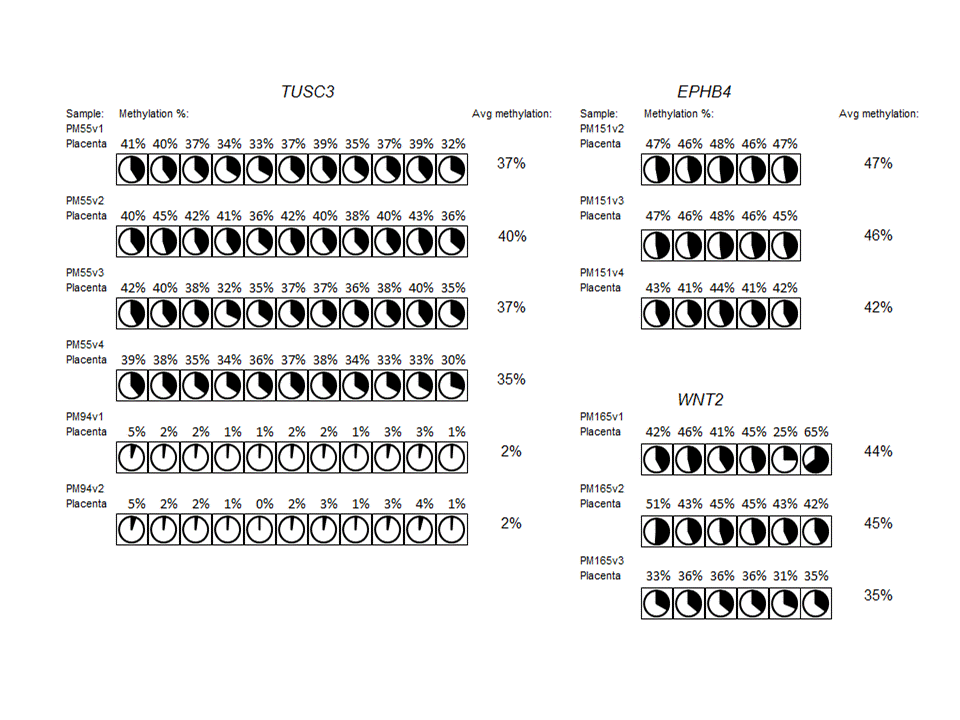

Supplement: Figure S2 — Intraindividual variability of DNA methylation on MAP. DNA methylation level of (A) TUSC3, (B) EPHB4 and (C) WNT2 is given for multiple whole villous samples taken from the same placenta (four sites for PM55, two sites for PM94). On-or-off DNA methylation pattern was consistent from different samples within the same placenta. Each circle represents a CpG site within a sample. The area shaded in black is proportional to the methylation level of the CpG site indicated by pyrosequencing. (0.08 MB GIF) [file pone.0007389.s002.gif]

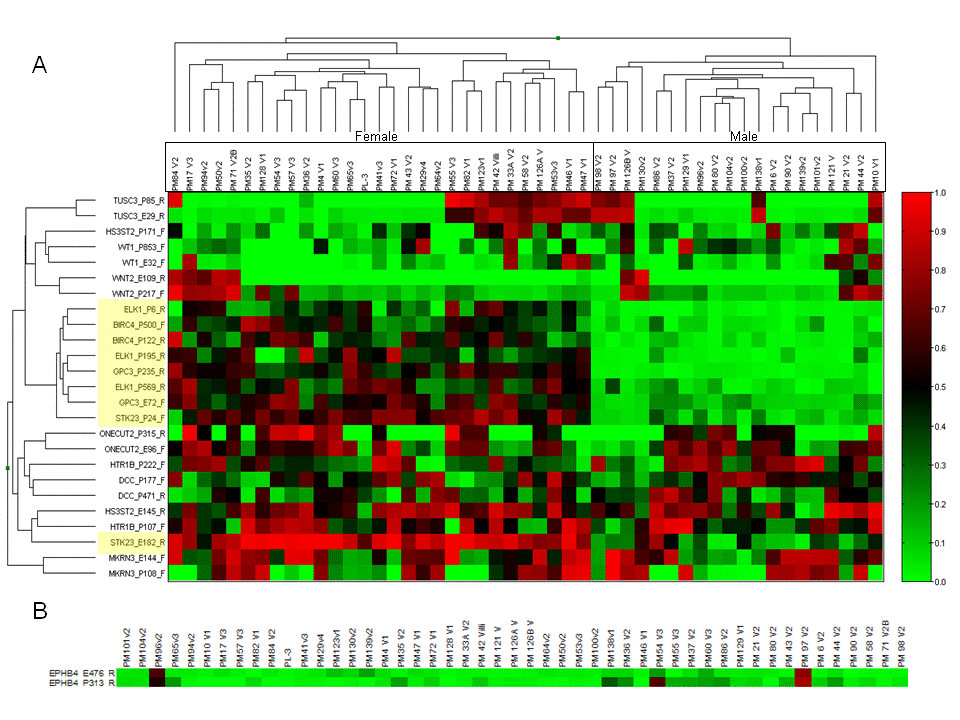

Supplement: Figure S3 — Genes exhibiting high interindividual variance in methylation values in a large population of human placentas. (A) Heat-map of 12 genes with at least 2 probes having methylation variance greater than 1.5 SD from the mean. Probes and sample names are shown and with hierarchical clustering of beta values based on 1-r (Illumina Beadarray software). A beta value of zero (indicated in bright green) represents an unmethylated locus and one (indicated in bright red) represents a methylated locus. Probes for genes on the X chromosome are highlighted by a yellow box. (B) Heat-map of EPHB4 in 49 human placentas. (0.14 MB GIF) [file pone.0007389.s003.gif]

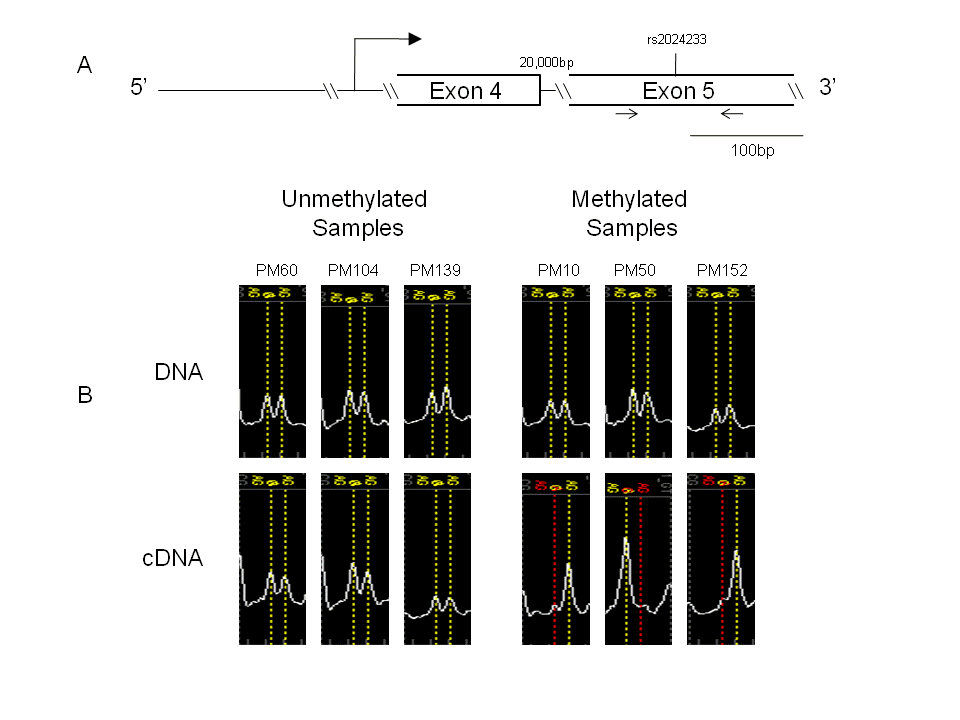

Supplement: Figure S4 — Allele-specific mRNA expression in WNT2. (A) Schematic of WNT2 locus showing the regions investigated by genotyping assays within exon 5 of 3 methylated samples and 3 unmethylated samples. PCR primers for DNA and cDNA genotyping by Sequenom are indicated by black arrows. (B) Allele-specific expression of WNT2 is observed based on the A/G allele of rs2228946 in DNA and cDNA by iPlex. Peak height of the alleles corresponds to the relative amount of alleles present in the sample. (0.04 MB GIF) [file pone.0007389.s004.gif]

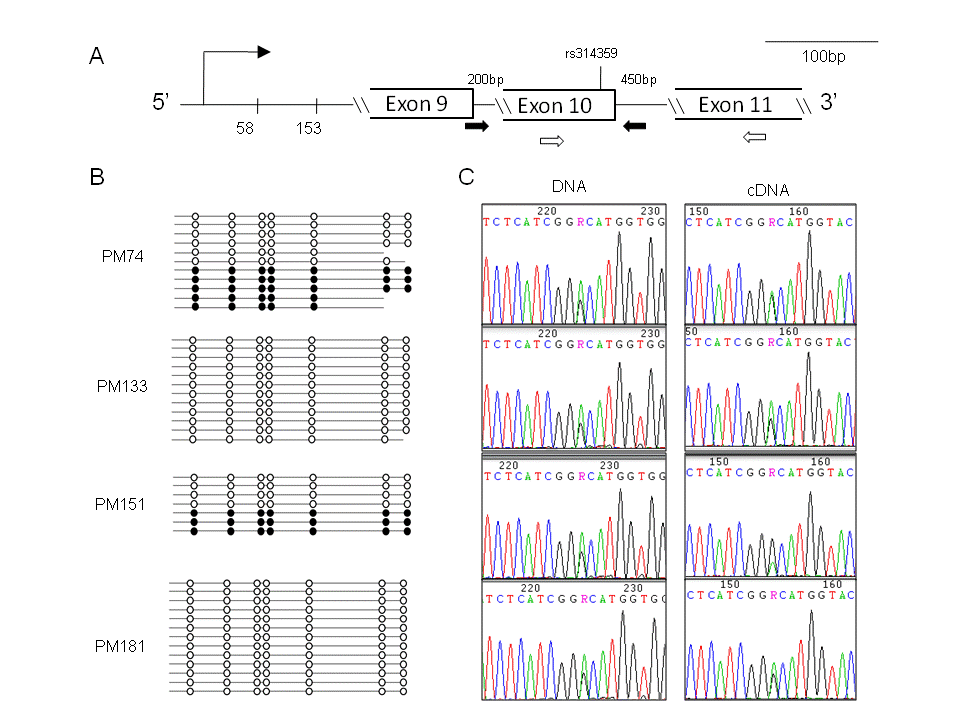

Supplement: Figure S5 — Allele-specific methylation and mRNA expression in EPHB4. (A) Schematic of the EPHB4 locus showing the regions investigated by clonal bisulfite pyrosequencing on the exon 1 (58 to 153 relative to the transcriptional start site according to NM_004444) and genotyping assays for exon 10. PCR primers for DNA by sequencing are indicated by black arrows while RT-PCR primers for mRNA genotyping are indicated by arrows highlighted in white. (B) Bisulfite pyrosequencing of single clones from four placenta samples. Each row represents one clone and each circle represents one CpG. Methylated CpGs are shown in black while unmethylated CpGs are shown in white. (C) Allele-specific expression of EPHB4 at A/G allele of rs314359 in DNA and cDNA by sequencing. Double peaks are shown in unmethylated samples. However, peaks on the SNP were skewed only in cDNA from methylated samples. (0.09 MB GIF) [file pone.0007389.s005.gif]

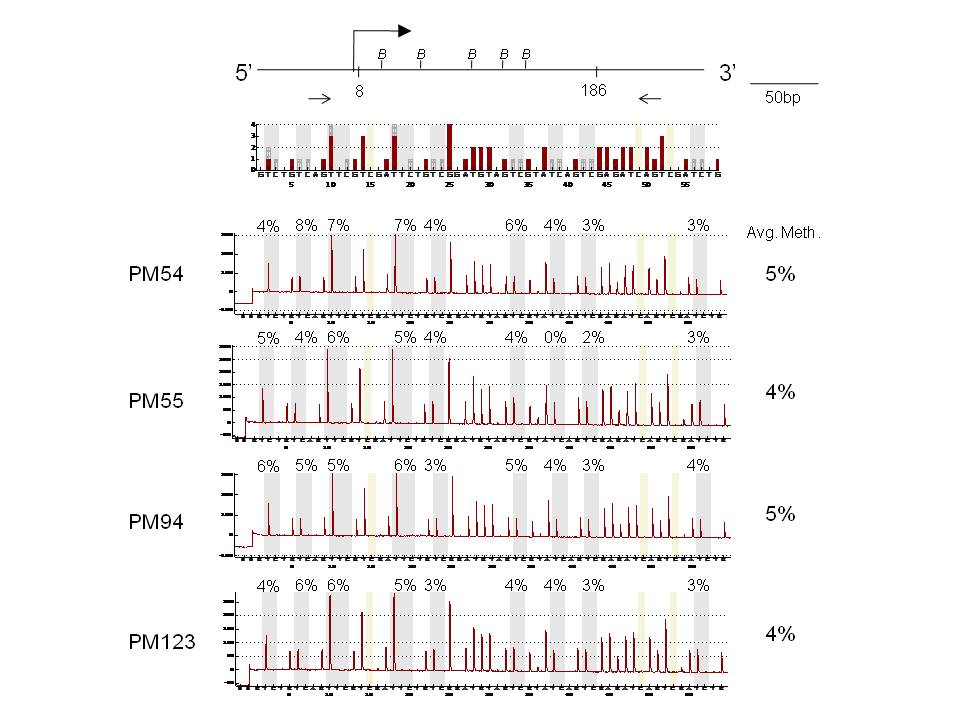

Supplement: Figure S6 — CpG methylation status of ID2 on BstUI digestion sites. Schematic of ID2 locus showing the regions investigated by bisulfite pyrosequencing is shown on top (8 to 186 relative to the transcriptional start site according to NM_002166). PCR primers for bisulfite pyrosequencing are indicated by black arrows. Enzyme digestion sites of BstUI are indicated by “B”. Reference pyrogram is provided. CpG sites were unmethylated for this region of ID2 in the 4 samples investigated. (0.03 MB GIF) [file pone.0007389.s006.gif]

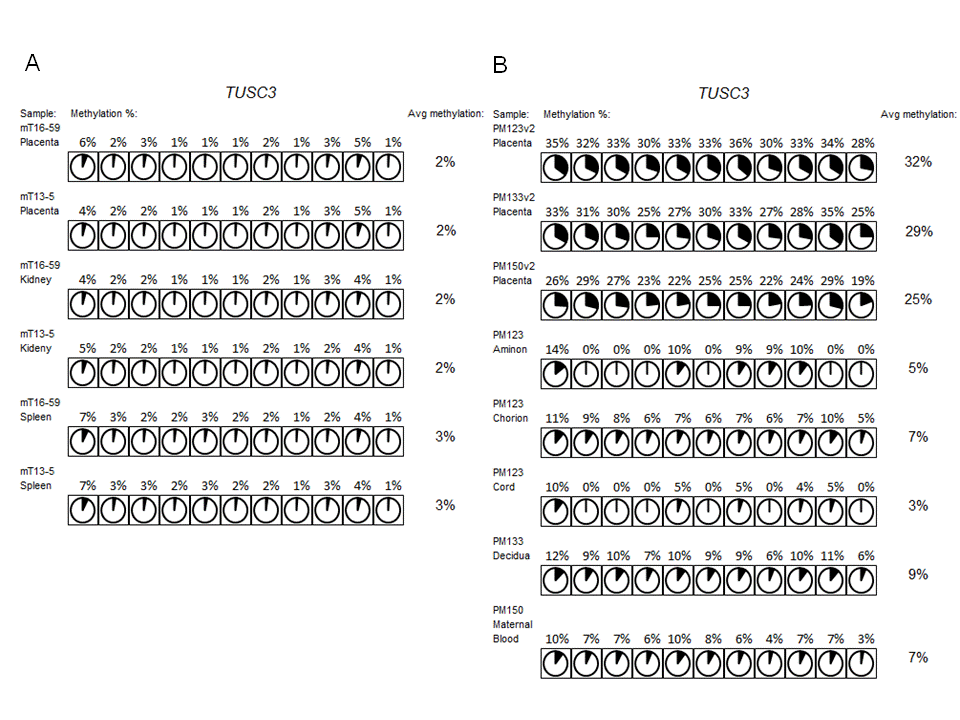

Supplement: Figure S7 — Tissue-specific DNA methylation of TUSC3. (A) DNA samples from two independent fetuses with placentas unmethylated in TUSC3 promoter were investigated by bisulfite pyrosequencing. None of the cases were methylated in other tissues. (B) DNA samples from 3 independent placentas with trophoblastic villi methylated in TUSC3 promoter were investigated by bisulfite pyrosequencing. None of the tissues (Aminon, chorion, cord, decidua and maternal blood) other than whole villi was highly methylated. Each circle represents a CpG site in a sample. Area shaded in black is proportional to the methylation level of the CpG site indicated by pyrosequencing. (0.12 MB GIF) [file pone.0007389.s007.gif]

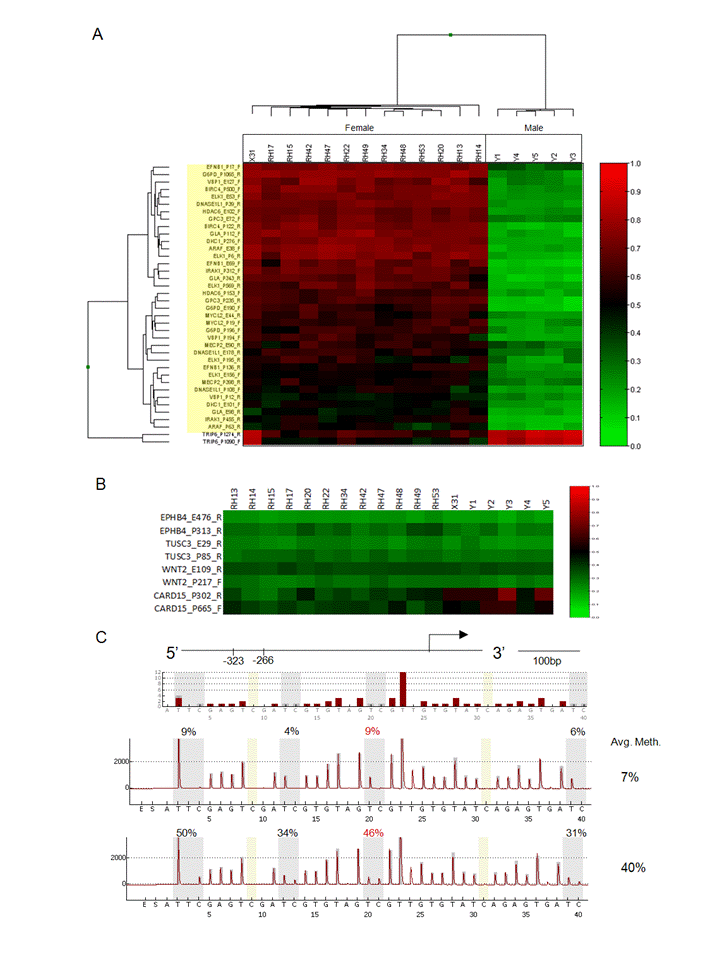

Supplement: Figure S8 — Interindividual variance of methylation values in the human blood cells. (A) Heat-map of 14 genes with at least 2 probes having methylation variance greater than 1.5 SD from the mean. Probes and sample names are shown and with hierarchical clustering of beta values based on 1-r (Illumina Beadarray software). A beta value of zero (indicated in bright green) represents an unmethylated locus and one (indicated in bright red) represents a methylated locus. Probes for genes on the X chromosome are highlighted by a yellow box. (B) Heat-map of EPHB4, TUSC3, WNT2 and NOD2 (CARD15) in human blood generated by Illumina GoldenGate Methylation Array. (C) Validation of variable methylation of NOD2 by bisulfite pyrosequencing. Schematic of NOD2 locus showing the regions investigated by bisulfite pyrosequencing (−323 to −266 relative to the transcriptional start site according to NM_022162). One methylated sample and one unmethylated sample are shown. Reference pyrograms are shown on top. Methylation level on CpG sites that targeted by the Illumina probe is highlighted in red. (0.10 MB GIF) [file pone.0007389.s008.gif]
